# Antioxidant capacity of honey from the urban apiary: a comparison with honey from the rural apiary

**DOI:** 10.1038/s41598-021-89178-4

**Published:** 2021-05-06

**Authors:** Agata W. Nicewicz, Łukasz Nicewicz, Patrycja Pawłowska

**Affiliations:** Research Team of Animal Physiology and Ecotoxicology, Faculty of Natural Sciences, Institute of Biology, Biotechnology and Environmental Protection, University of Silesia, Bankowa 9, 40-007 Katowice, Poland

**Keywords:** Biomarkers, Environmental impact, Natural hazards

## Abstract

Honey is a source of natural antioxidant compounds exerting several health-beneficial effects. Since urban beekeeping is quite common, the fear among potential consumers about the quality and the safety of honey produced exclusively in the cities is observed. However, the antioxidant properties of urban honey have not yet been tested. We described the antioxidant properties of linden honey from urban and rural areas. We analyzed the total phenolic content, DPPH^•^ radical scavenging activity, Trolox equivalent antioxidant activity assay, the protein content, and catalase activity. The analysis showed that all tested parameters were significantly higher in honey from rural areas than in urban samples. The differences in the obtained results are certainly not the effect of the floral composition of honey, but rather due to the location of the honeybee colonies. It seems that the consumption of honey from urban areas for health purposes should be considered.

## Introduction

Honey, a remarkably complex natural liquid, can exert several health-beneficial effects such as antibacterial, anti-fungal, and anti-inflammatory effects^1^. One of the most valuable effects of honey on human health is its antioxidant properties. Studies to date have shown that these honey properties help manage chronic diseases commonly associated with oxidative stress. Antioxidant compounds from honey can prevent cancer, enhance the immune response, positive effect on infectious diseases, prevention of aging, and as cardioprotective agents^1,2^. Honey contains several antioxidant compounds, including organic acids, flavonoids, phenolic compounds, carotenoid derivatives, enzymes (catalase, glucose oxidase), and vitamins (ascorbic acid, tocopherols), and amino acids^3^.

Urban beekeeping is nowadays quite common and is a viable source of income^4^. However, the localization of hives raised fear among potential consumers about the quality and the safety of honey produced exclusively in the cities. Several studies have dispelled these doubts (e.g.^5^). Honey from urban apiaries is not more polluted by heavy metals than honey from agricultural areas. Interestingly, honey's antioxidant properties from urban apiaries have not yet been deep tested, although they are important from the point of view of a potential consumer.

## Results

### Melissopalinological analyses

**Figure 1:**
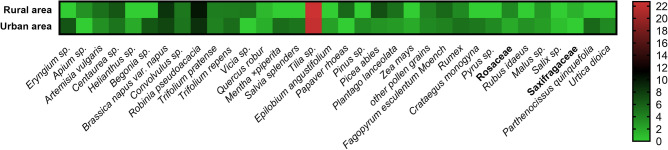
Pollen types obtained from the honey samples and their frequency. Heatmap of the mean frequency of pollens in honey samples from urban and rural areas.

The average pollen content of honey samples in two different apiaries localisations is depicted in Fig. 1 with the scientific names and pollen frequency. Table 1 summarizes the average prevalence of dominant pollen from several plant species (pollen frequency over 6%). The highest mean pollen content for all honey samples was found for linden (*Tillia sp.*) pollen regardless of the location. The average linden pollen frequency was 22.0 ± 1.71 for samples from the urban and agricultural areas—22.74 ± 1.05 (Table 1). Pollen from acacia (*Robinia pseudoacacia*), rape (*Brassica napus var. napus*), and cornflowers (*Centaurea sp.*) was also found in samples from both locations. In addition to the dominant pollen from 6 species, pollen from 28 different plant species, including wind-pollinated, was found. Among these species, grass pollen (a separate category), corn (*Zea mays*), pine (*Pinus sp.*), spruce (*Picea abies*), oak (*Quercus robur*), and willow (*Salix sp.*) pollen were identified (Fig. 1).

**Table 1:** The prevalence of the dominant pollen of several plant species.

Plant species	Localizations	
	Rural areas	Urban areas
*Tilia sp.*	22.74 ± 1.05^**a**^	22.0 ± 1.71^**a**^
*Robinia pseudoacacia*	10.12 ± 5.3^**a**^	8.68 ± 3.09^**a**^
*Brassica napus var. napus*	8.43 ± 3.14^**a**^	7.44 ± 1.54^**a**^
*Eryngium sp.*	nb	6.69 ± 1.72
*Begonia sp.*	nb	6.6 ± 0.23
*Centaurea sp.*	6.56 ± 2.11^**a**^	4.89 ± 1.62^**a**^

The pollen [mean ± SD] frequency from few species of plants in honey samples from urban and rural apiaries. Only a few dominant plant species (pollen frequency over 6%) are presented. Different letters—significant differences between pollen frequency in honey samples from two types of the apiary. Student t-test. *p* ≤ 0.05.

*nb* not observed.

### Physiochemical parameters

The average moisture content of honey samples was statistically different depending on the apiary localization and was 15.42 ± 1.02 for samples from the urban area and agricultural areas—16.69 ± 0.53 (Table 2).

**Table 2:** Physicochemical properties of analyzed honey samples.

Sample no	Moisture (%)	pH	Electrical conductivity (mS/cm)
U1	14.1	4.5	0.48
U2	16	4.25	0.62
U3	16.1	4.11	0.61
U4	14.0	4.12	0.52
U5	14.1	4.7	0.58
U6	15.0	4.55	0.6
U7	15.0	4.01	0.54
U8	16.9	3.99	0.56
U9	15.3	4.15	0.55
U10	15.9	4.6	0.48
U11	18.1	3.98	0.55
U12	16.9	4.01	0.56
U13	15.5	3.85	0.55
U14	14.0	3.46	0.48
U15	14.8	3.88	0.61
U16	16.2	3.55	0.55
U17	14.8	4.5	0.55
U18	14.8	4.05	0.48
U19	15.0	3.85	0.54
U20	14.9	3.66	0.55
U21	15.9	4.05	0.50
U22	14.9	4.15	0.57
U23	14.3	4.25	0.55
U24	14.0	4.1	0.52
U25	16.7	4.0	0.49
U26	16.0	3.89	0.55
U27	16.0	4.25	0.61
U28	16.5	4.3	0.58
U29	15.8	3.75	0.62
U30	15.0	3.75	0.55
Average	15.42 ± 1.02^a^	4.08 ± 0.3^a^	0.55 ± 0.04^a^
R1	15.8	3.82	0.52
R2	16.3	4.01	0.60
R3	16.4	4.01	0.55
R4	17.5	3.58	0.58
R5	15.7	3.89	0.50
R6	16.2	4.15	0.54
R7	17.1	4.05	0.61
R8	17.1	3.99	0.55
R9	17.1	4.00	0.50
R10	18.0	4.01	0.53
R11	16.9	3.78	0.61
R12	16.0	4.3	0.56
R13	16.5	4.22	0.48
R14	16.5	3.78	0.52
R15	16.9	4.22	0.56
R16	17.0	4.11	0.54
R17	16.6	4.01	0.48
R18	16.0	3.78	0.42
R19	16.3	4.11	0.48
R20	17.5	4.35	0.51
R21	17.2	4.05	0.51
R22	16.9	3.78	0.52
R23	16.5	3.99	0.55
R24	16.8	4.05	0.55
R25	17.0	4.12	0.55
R26	16.3	3.78	0.48
R27	16.8	3.89	0.58
R28	16.2	3.89	0.57
R29	17.0	4.25	0.51
R30	16.5	4.12	0.55
Average	16.69 ± 0.53^b^	4.00 ± 0.15^a^	0.53 ± 0.04^a^

Physicochemical properties of all analyzed honey samples [mean ± SD] from urban and rural apiaries. Mean values for each parameter and localization with different letters differ significantly. Student t-test. *p* ≤ 0.05.

Samples origin from *U* urban and *R* rural apiaries.

The average electrical conductivity was 0.55 ± 0.04 mS/cm for honey samples from urban areas and 0.53 ± 0.04 mS/cm for samples from rural areas (Table 2). Statistically difference in the honey electrical conductivity between samples from two localisations was not observed.

The average pH of honey samples was not statistically different depending on the apiary localization and was 4.08 ± 0.3 for samples from the urban area and agricultural areas—4.00 ± 0.15 (Table 2).

### Antioxidant properties

**Figure 2:**
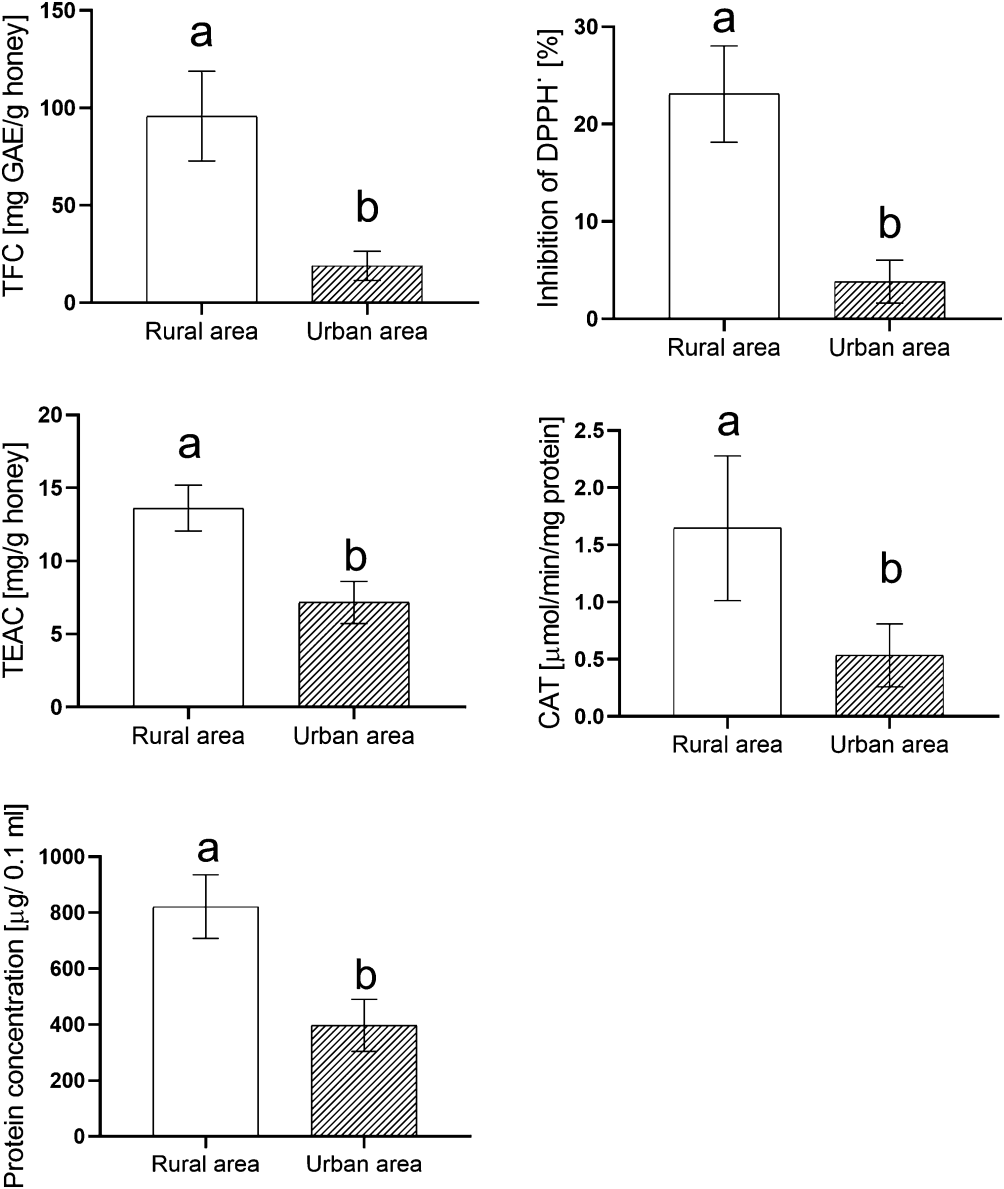
Comparison of antioxidant properties of honey from urban and rural areas. TPC (total phenolic content) is expressed as mg gallic acid equivalents per 100 g of honey sample (mgGAE/100 g). RSA (DPPH^•^ radical scavenging activity) is expressed as a percent of inhibition of DPPH^•^ radical. TEAC (Trolox equivalent antioxidant activity) is expressed as mg of Trolox equivalents per 100 g of honey. Catalase activity is shown as µmol H2O2 per min per mg protein (µmol/min/mg protein). The protein content is expressed as µg protein per 100 µl of honey solution. All values are presented as a mean ± SD. Different letters—significant differences. Student t-test. *p* ≤ 0.05.

Values of tested parameters indicating the antioxidant properties of honey were statistically significantly higher in honey samples from rural areas. Samples from the rural landscape had an eightfold higher TFC than honey from the urban are (Fig. 2). The total antioxidant capacity of samples was also measurement. The RSA expressed as % inhibition of DPPH• radical by honey was sixfold higher in samples from rural areas (23% in comparison with 3.8% of urban honey). The TEAC expressed as mg of Trolox equivalents per 100 g of honey was also less in honey from the urban area (twofold) than in rural samples (Fig. 2). No statistically significant correlation was observed between TFC and RSA (urban honey—*r* = 0.297, *p* = 0.11, rural area—*r* = 0.06, *p* = 0.731) and TFC and TEAC (urban honey—*r* = 0.152, *p* = 0.42, rural area—*r* = 0.06, *p* = 0.752).

The protein concentration was twice higher in the linden honey from rural areas than in urban products (Fig. 2). No statistically significant correlation was observed between protein content and radical-scavenging activity in honey (urban honey—*r* = 0.085, *p* = 0.657, rural honey—*r* = 0.081, *p* = 0.672).

The catalase activity was threefold higher in the honey from the rural site (Fig. 2).

## Discussion

Pollen analysis (Fig. 1, Table 1) was used to specifically identify and confirm the botanical origin of honey samples from rural and urban localisations. Linden honey is included in the undistrushed honey, which comes from plants with a low pollen yield or plants with flowers that have a structure that makes it difficult to dust the nectar with pollen. Therefore, it is assumed that the proportion of main linden pollen in this kind of honey should be at least 20%, while e.g. in the case of rapeseed honey, the proportion of rapeseed pollen should be min. 45%^6,7^.

Melissopalinological analysis confirmed the organoleptic test that all analyzed samples from both areas are linden honey (linden pollen content minimum 20%) (Table 1). In the tested samples, the presence of pollen grains from 34 plant species (Fig. 1), including wind-pollinated plants, was demonstrated. These plants bloomed at the same time as linden and present in urban flowerbeds, wastelands, meadows, or green belts between fields e.g., cornflowers (*Centaurea sp.*), poppies (*Papaver rhoeas*), virginia creeper (*Parthenocissus quinquefolia*), eryngo (*Eryngium sp.*), mint (*Mentha* × *piperita*). Pollen of some plants may have appeared in honey by accident (contaminated by bees in the hive or by a beekeeper, e.g., rape pollen). However, the dominant pollen in the samples was Tilia pollen. The pollen of other plant species was characterized by a lower frequency of occurrence (10% or less).

The honey moisture content depends mainly on the time of harvest, environmental conditions, and the manipulation by beekeepers. Low moisture content values indicate for the honey ripe and increase its shelf life and the storage time through prevention fermentation^8,9^. Therefore according to international regulations^10,11^, the moisture content in honey should be below 20%. The moisture content in the tested samples ranged from 14.0 to 17.5%, which are within the allowed parameters (below 20%) according to the international regulations of quality^10,11^. Differences in water content in honey samples may result, for example, from temperature differences in the location of the apiary. Hives from urban apiaries are usually located on the roofs of buildings, which favors the increase in the already high temperature in the city (heat island effect)^12^. Higher temperature tends to the evaporation of water during honey maturation in bee colonies.

The electrical conductivity is a good criterion of the botanical and geographical origin of honey. Its level depends on the ash and acid content of honey, and these depend on the high mineral content of the soil^8^. This parameter also indicates for the type of honey (blossom honey or honeydew)^6^. The electrical conductivity values of all tested samples were within the standard limit (lower than 0.8 mS/cm)^10,11^ and the values in honey samples varied in the range of 0.42–0.62 mS/cm (Table 1). The obtained results indicate that all analyzed samples were blossom honey and not honeydew^6^.

The high acidity is a characteristic feature of multiflorous, linden, and rapeseed types of honey^9^. All tested samples were found to have an acidic character. Their pH values ranged from pH 3.46 to pH 4.25 (Table 1).

The obtained results of physicochemical parameters are consistent with the literature data about linden honey: honey moisture content in the range 15.20–19.32%^13^, the electrical conductivity in the range 0.46–0.54 mS/cm^13–16^, and the pH in the range 3.74–5.4^13–16^.

Currently, the antioxidant properties of honey are considered an important quality parameter^17^. This parameter is strongly correlated to phenolic substances, efficient oxygen radical scavengers, derived from nectar and pollen^9^. Some phenolic substances are tested as a component of pharmaceutical drugs against cancer. Honey consumption is recommended for supporting and mobilizing the immune system^2^. Therefore, honey antioxidant capacity is crucial for potential consumers. Thus, the few parameters indicating the antioxidant properties of honey were investigated. Total phenolic content, DPPH• radical scavenging activity (RSA), and Trolox equivalent antioxidant activity (TEAC) were significantly higher in honey samples from rural areas.

Previous studies have shown that the chemical composition of honey primarily depends on the botanical origin. Moreover, the variety of flower resources is also important, not just the abundance of one particular species. It seems that this is an important issue in the case of urban honey. Recently, Kavanagh et al.^8^ revealed that TFC level was significantly higher in the honey samples from urban areas of Ireland than from rural landscapes. As an explanation of the obtained results, Kavanah et al.^8^ have just pointed to the different botanical origins of the compared kinds of honey and the higher floral resource diversity in some urban landscapes. This explanation also shows why we obtained the opposite results. In our study compared linden honey samples, so the differences in the botanical origin and diversity of the floral resources of the honey cannot explain our results.

Climatic conditions, seasonal and/or geographical origin can also impact the chemical composition of even within the same type of honey (e.g.^9,18–20^). TFC and their antioxidant activity are particularly strongly dependent on the specific climatic conditions and plant sources^17^. However, these factors do not explain the differences we found. Samples were originally from the same time in the season, from the same climatic zone, the honey type was the same (linden honey), and the distance between the apiaries was only 100 km. The source of variability in the obtained results is, therefore, the local environmental factors as Alvarez-Suarez et al.^9^ and Soares et al.^20^ indicated. Two different locations of apiaries—the center of a large city (concrete heat island) and a typical polish agricultural landscape, are two different ecosystems influenced by different factors, including local microclimate^12^.

The antioxidant compounds in honey come mainly from nectar and pollen^21^. Plants synthesize phenolic compounds during normal development (basic phenolic compounds) and as a result of stress caused by biotic and abiotic factors^22^. The abiotic factors that affect the polyphenol content in the plant include the climate, soil, and agrotechnical conditions of cultivation.

Differences shown in our study may just result from the variety in abiotic factors characterizing the location of the tested apiaries. Rural apiary is located in the agricultural landscape typical for this region of Poland without large-area monoculture crops, with a small share of artificial fertilizers but with the commonly used natural fertilizer–manure. Research shows that the accumulation of natural fertilizers in the soil has a positive effect on the content of polyphenols in plants^22,23^. On the other hand, fertilization in urban areas is limited only to small areas of flower beds and is based on artificial fertilizers.

Air temperature and its daily fluctuations, air humidity, and water availability can also play a crucial role in the content of polyphenols in plants and thus in the nectar and pollen. In the rural landscape the large daily temperature fluctuations, strong sunlight, and limited water availability (average annual rainfall—600 mm; Climate-Data.org) are observed. On the other hand, the air temperature in the city is higher, but not strongly fluctuated (heat-island effect^24^), the availability of water is facilitated (the presence of the river—Rawa, the average annual rainfall—689 mm; Climate-Data.org) and the air humidity is higher than in rural areas^25,26^. Research shows that plants subjected to drought stress synthesize large amounts of polyphenols^22^.

An essential factor forming the content of polyphenols in plant tissues is soil salinity^22^. In polluted areas with the mining and metallurgic industry, the salinity of urban soil increases^26^, which can be leading to a decrease in the content of polyphenols in the tissues^22^. This may be one of the many reasons for the weak antioxidant properties of the analyzed urban honey samples.

Our results about the correlation between TFC and RSA and TEAC indicate that the phenolic compounds are not the main contributor to the TFC of tested honey. The lack of this relationship is contrary to the literature data (e.g.^9,17^).

The honey protein level is dependent on the type of flora. The different protein content is based on the presence of enzymes introduced by bees themselves and others derived from the nectar^9^. Our results revealed that the protein concentration was twice higher in the linden honey from rural areas than in urban products (Fig. 2). It is known that the antioxidant capacity of honey is the result of the wide range of compounds activity, e.g., organic acids, phenolic compounds, and also proteins. However, the protein fraction of honey from both areas (enzymes, amino acids, and peptides) does not contribute to its antioxidant capacity since no statistically significant correlation was observed between protein content and radical-scavenging activity honey. Alvarez-Suarez et al.^9^ reported similar results. Other data indicate that proline content and not total protein level could play a role in its antioxidant activity^27^.

Catalase is the enzyme naturally present in honey. The source of the enzyme is pollen, nectar, yeasts, and other microorganisms. Catalase controls the H_2_O_2_ balance in the honey by regulating glucose-oxidase activity. Although catalase is little studied their role can be important for example to the success of honey-dressings with antimicrobial properties^28^. Our results revealed the catalase activity was significantly higher in the honey from the rural site (threefold) (Fig. 2).

## Conclusions

Our research revealed crucial differences in honey's antioxidant properties from locations differing in the degree of urbanization and industrialization. The total phenolic content, total antioxidant capacity (RSA, TEAC), protein content, and catalase activity were significantly higher in honey from rural areas than in urban samples. The differences in the obtained results are certainly not the effect of the floral composition of honey, but rather due to the honeybee colonies' location. To our knowledge, this is the first comparison of the antioxidant properties of honey with the same botanical origin from the typical urban and rural apiaries. Our findings suggest that the consumption of honey from urban areas for health purposes should be considered.

## Materials and methods

Linden honey samples were collected from hives located in areas that differ in degree of urbanization and industrialization. These areas are described by the new degree classification of urbanization defined according to the population size, density and contiguity of local administrative units level 2 (LAU2) by the European Commission^29,30^. Honey was provided by the apiaries run by the University of Silesia. The urban apiary is located on the rooftop of a 30-m high building in the center of Katowice city (50.2649° N, 19.0238° E) and surrounded mainly by office towers and apartment blocks. The apiary was provided Rural honey in a typical polish agricultural landscape, with low-area crops and pesticides in the Parzymiechy village (51.0401° N, 18.7376° E).

Samples were collected according to Matin et al.^31^. The qualitative microscopic analysis of pollen determined the kind of honey according to Polish Standard (PN-88/A-77626, 1998) based on the work by Louveaux et al.^32^. A total of 30 samples from each localization was gathered. All collected samples were stored in the fridge between 0 and 4 °C before analysis^27,33^.

The tested honey was characterized in terms of its physicochemical properties. The moisture content of samples was measured according to the Association of Official Analytical Chemists (AOAC) method^34^, and results were expressed as percentages. The pH of tested honey samples was determined according to methods of the International Honey Commission^35^. Electrical conductivity (EC) was measured according to the harmonized methods of the European Honey Commission^35^. Results were expressed as millisiemens per centimeter (mS/cm).

The following parameters have been determined: total phenolic content (TPC) using a modified Folin-Ciocalteu method^27,36^, DPPH^•^ radical scavenging activity (RSA)^37^, Trolox equivalent antioxidant activity (TEAC) assay^38^, the protein content^39^, catalase activity^40^.

TPC results were expressed as mg of gallic acid equivalents (GAE, Sigma Aldrich) per 100 g of honey basing on a six-point calibration curve of gallic acid (Sigma Aldrich)^27^. RSA (DPPH• radical scavenging activity) was expressed as a percent of inhibition of DPPH• radical (Sigma Aldrich). TEAC (Trolox equivalent antioxidant activity) was expressed as mg of Trolox (Calbiochem) equivalents per 100 g of honey with a curve between 0.05 and 0.25 µg/ml (*r*^2^ ≥ 0.99). Catalase activity is shown as µmol H_2_O_2_ (Sigma Aldrich) per min per mg protein (µmol/min/mg protein). The protein content is expressed as µg protein per 100 µl of honey solution calculated based on the bovine serum albumin (BSA, Sigma Aldrich) series solution as a protein standard to the curve creation in the range of 0–1000 ug/ml. The linear calibration and the coefficient of determination (*r*^2^) were analyzed. A value of *r*^2^ greater than 0.99 was considered satisfactory.

All analytic determinations were carried out in triplicate and the data were expressed as means ± SD. Normality was checked with the Kolmogorov–Smirnov test. Student's t-tests were performed to determine differences (*p* ≤ 0.05) in the analyzed parameters between the two tested locations (city vs. village). Correlations were obtained by Pearson's correlations between TPC, RSA, TEAC, protein content and catalase activity. GraphPad Prism (GraphPad Software Inc, San Diego, CA, USA) software was used for statistical calculations.

## Data Availability

The datasets generated during and/or analysed during the current study are available from the corresponding author on reasonable request.
